# P-267. Methodological Framework for Estimating Infections Prevented by Infection Control Services

**DOI:** 10.1093/ofid/ofae631.471

**Published:** 2025-01-29

**Authors:** Bráulio R G M Couto, Raquel Bandeira, Gabrielle R Mota, Fernanda Santos, Rossana Souza, Ana Paula Ladeira, Pamela Leite, Lívia Miranda

**Affiliations:** AMECI – Associação Mineira de Epidemiologia e Controle de Infecções, Belo Horizonte, Minas Gerais, Brazil; Hospital Metropolitana Doutor Célio de Castro, Belo Horizonte, Minas Gerais, Brazil; Hospital Universitário Ciências Médicas (HUCM), Belo Horizonte, Minas Gerais, Brazil; Hospital Universitário Ciências Médicas (HUCM), Belo Horizonte, Minas Gerais, Brazil; Biobyte Sistemas, Belo Horizonte, Minas Gerais, Brazil; Biobyte Tecnologia em Epidemiologia, Belo Horizonte, Minas Gerais, Brazil; Hospital Universitário Ciências Médicas (HUCM), Belo Horizonte, Minas Gerais, Brazil; Faculdade Dinâmica Vale do Piranga - FADIP, Viçosa, Minas Gerais, Brazil

## Abstract

**Background:**

This report aims to propose a methodology for estimating the number of infections prevented by an Infection Control Service, presenting observed outcome estimates at a University Hospital in 2023.

**Table 1:**
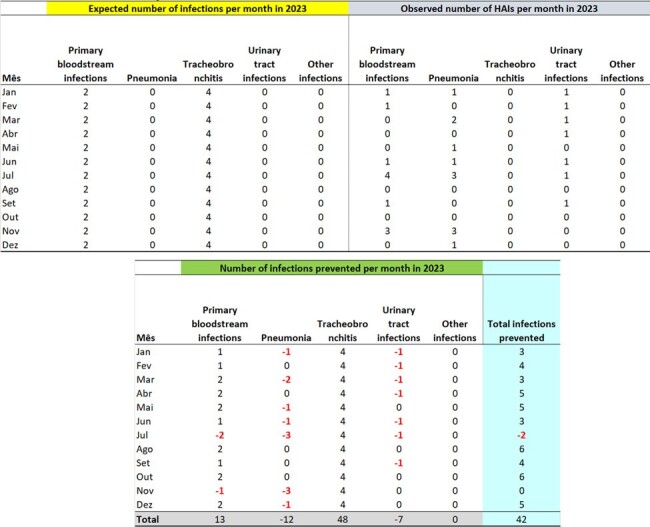
Expected Hospital Infections for 2023, Observed Infections, and Infections Prevented in 2023, Month-by-Month, for the ICU on the 3rd Floor Expected Hospital Infections for 2023, Observed Infections, and Infections Prevented in 2023, Month-by-Month, for the ICU on the 3rd Floor

**Methods:**

Prevented infection is estimated by the Infectometer, a real-time system that provides information on the expected weekly and monthly incidence of specific infections. To estimate the number of infections prevented each year, the expected monthly HAI count is calculated based on endemic curves from the immediately preceding year. This figure is then compared with the actual number of infections observed month-to-month, yielding an estimate of the number of infections avoided. To calculate Infectometer parameters: (1) construct the Statistical Process Control (SPC) for each infection or microorganism; (2) estimate the average month (m) and standard deviation (s) of the infection cases, excluding outlier data, i.e., data from months with rates higher than the maximum control limit; (3) calculate the Monthly Expected Incidence (MEI), assuming that the incidence of new infections (NI) follows a normal distribution. The 50th percentile from the normal distribution is used when the objective is to estimate the number of prevented HAIs.

**Table 2:**
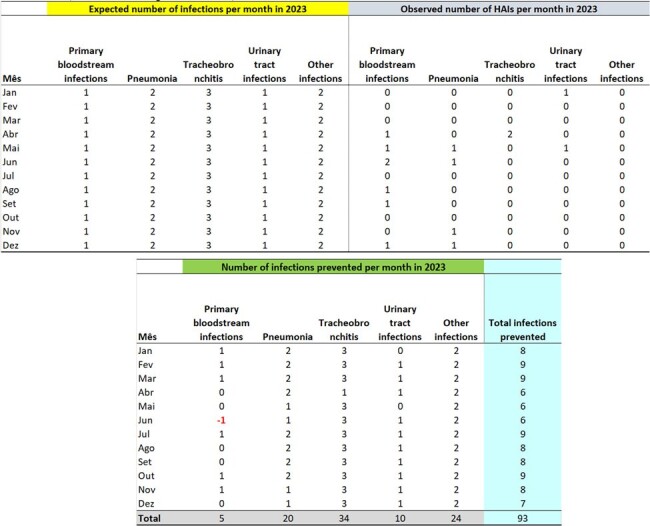
Expected Hospital Infections for 2023, Observed Infections, and Infections Prevented in 2023, Month-by-Month, for the ICU on the 1st Floor Expected Hospital Infections for 2023, Observed Infections, and Infections Prevented in 2023, Month-by-Month, for the ICU on the 1st Floor

**Results:**

The number of expected infections per month in 2023, by site, was estimated based on the Infectometer and parameters from the previous year (2022), with 13 surgical site infections, 12 tracheobronchitis, 7 urinary tract infections, 6 primary bloodstream infections, 6 pneumonia, and 4 other infections. Based on the number of infections observed in 2023, there were 43 infections on the 2nd Floor, 128 on the 4th Floor, 93 in the ICU on the 1st Floor, and 42 in the ICU on the 3rd Floor, totaling 306 infections prevented.

**Table 3:**
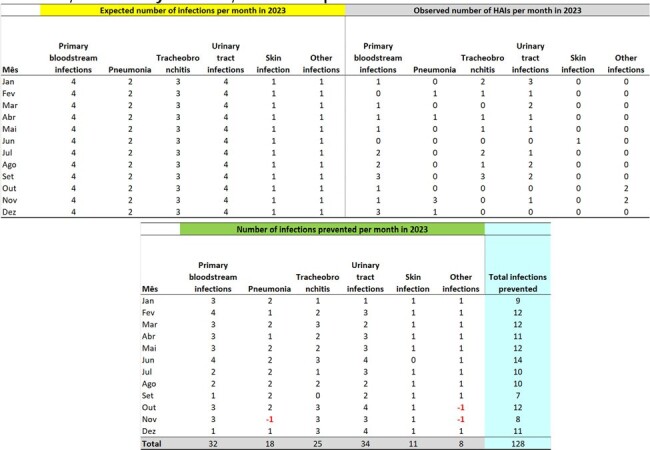
Expected Hospital Infections for 2023, Observed Infections, and Infections Prevented in 2023, Month-by-Month, for the Inpatient Units on the 4th Floor Expected Hospital Infections for 2023, Observed Infections, and Infections Prevented in 2023, Month-by-Month, for the Inpatient Units on the 4th Floor

**Conclusion:**

A methodology was presented for estimating the number of infections prevented by the actions of a multimodal, multifaceted, and multiprofessional program managed by the Infection Control Service (ICS), whose main objective is to control and prevent healthcare-associated infections (HAIs) at a university hospital. Based on this methodology, 306 infection cases were prevented in 2023, saving at least $125,154 over one year. Additionally, 70 deaths were prevented in 2023.

**Table 4:**
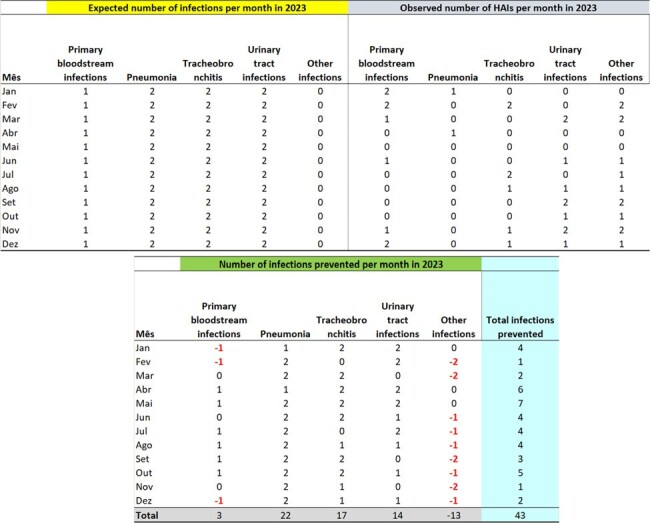
Expected Hospital Infections for 2023, Observed Infections, and Infections Prevented in 2023, Month-by-Month, for the Inpatient Units on the 2nd Floor Expected Hospital Infections for 2023, Observed Infections, and Infections Prevented in 2023, Month-by-Month, for the Inpatient Units on the 2nd Floor

**Disclosures:**

**All Authors**: No reported disclosures

